# Diversity of *Salmonella* spp. serovars isolated from the intestines of water buffalo calves with gastroenteritis

**DOI:** 10.1186/1746-6148-8-201

**Published:** 2012-10-25

**Authors:** Giorgia Borriello, Maria G Lucibelli, Michele Pesciaroli, Maria R Carullo, Caterina Graziani, Serena Ammendola, Andrea Battistoni, Danilo Ercolini, Paolo Pasquali, Giorgio Galiero

**Affiliations:** 1 Istituto Zooprofilattico Sperimentale del Mezzogiorno, Via Salute, 2, 80055, Portici, Italy; 2 Istituto Superiore di Sanità, Dipartimento di Sanità Pubblica Veterinaria e Sicurezza Alimentare, Viale Regina Elena, 299, 00161, Rome, Italy; 3 Università di Roma Tor Vergata, Dipartimento di Biologia, via della Ricerca Scientifica, 1, 00133, Rome, Italy; 4 Dipartimento di Scienza degli Alimenti, Università degli Studi di Napoli Federico II, Via Università 100, 80055, Portici, Italy

**Keywords:** *Salmonella*, Virulence markers, Genetic characterization, Gastrointestinal ecology

## Abstract

**Background:**

Salmonellosis in water buffalo (*Bubalus bubalis*) calves is a widespread disease characterized by severe gastrointestinal lesions, profuse diarrhea and severe dehydration, occasionally exhibiting a systemic course. Several *Salmonella* serovars seem to be able to infect water buffalo, but *Salmonella* isolates collected from this animal species have been poorly characterized. In the present study, the prevalence of *Salmonella* spp. in water buffalo calves affected by lethal gastroenteritis was assessed, and a polyphasic characterization of isolated strains of *S*. Typhimurium was performed.

**Results:**

The microbiological analysis of the intestinal contents obtained from 248 water buffalo calves affected by lethal gastroenteritis exhibited a significant prevalence of *Salmonella* spp. (25%), characterized by different serovars, most frequently Typhimurium (21%), Muenster (11%), and Give (11%). The 13 *S*. Typhimurium isolates were all associated with enterocolitis characterized by severe damage of the intestine, and only sporadically isolated with another possible causative agent responsible for gastroenteritis, such as *Cryptosporidium* spp., Rotavirus or *Clostridium perfringens*. Other *Salmonella* isolates were mostly isolated from minor intestinal lesions, and often (78% of cases) isolated with other microorganisms, mainly toxinogenic *Escherichia coli* (35%), *Cryptosporidium* spp. (20%) and Rotavirus (10%). The *S*. Typhimurium strains were characterized by phage typing and further genotyped by polymerase chain reaction (PCR) detection of 24 virulence genes. The isolates exhibited nine different phage types and 10 different genetic profiles. Three monophasic *S*. Typhimurium (B:4,12:i:-) isolates were also found and characterized, displaying three different phage types and three different virulotypes. The molecular characterization was extended to the 7 *S*. Muenster and 7 *S*. Give isolates collected, indicating the existence of different virulotypes also within these serovars. Three representative strains of *S*. Typhimurium were tested *in vivo* in a mouse model of mixed infection. The most pathogenic strain was characterized by a high number of virulence factors and the presence of the locus *agfA*, coding for a thin aggregative fimbria.

**Conclusions:**

These results provide evidence that *Salmonella* is frequently associated with gastroenteritis in water buffalo calves, particularly *S*. Typhimurium. Moreover, the variety in the number and distribution of different virulence markers among the collected *S*. Typhimurium strains suggests that within this serovar there are different pathotypes potentially responsible for different clinical syndromes.

## Background

*Salmonella* spp. found in water buffalo (*Bubalus bubalis*) herds are a matter of concern since they are responsible for serious economic losses in livestock and are a zoonotic agent responsible for foodborne illness [1]. As for bovine calves, *Salmonella*-induced diseases in water buffalo calves are characterized by severe gastrointestinal lesions, profuse diarrhea, and severe dehydration [1]. Acute salmonellosis generally induces diarrhea, mucous at first, later becoming bloody and fibrinous, often containing epithelial casts. Ingestion is the main route of infection, although it can also occur through the mucosa of the upper respiratory tract and conjunctiva. The major source of infection in the herd is represented by asymptomatic older animals shedding heavy loads of bacteria through feces. Other sources of infection are contaminated forages and water, as well as rodents, wild winged animals, insects and man [1,2]. The disease can also cause sudden death without symptoms. Occasionally, the infection is systemic, affecting joints, lungs and/or the central nervous system (CNS) [1]. Moreover, several *Salmonella* serovars seem to be able to infect water buffalo, mainly affecting 1–12 week old calves, even though reports on salmonellosis in *B*. *bubalis* are scarce [1,3].

Water buffalo calves are more frequently affected by gastroenteritis than bovine calves, with mortality rates as high as 70% in water buffalo species vs. 50% in bovine [1,4]. This difference might be due to a greater susceptibility of water buffalo to gastroenteric pathogens, although it also may reflect the lack of appropriate management practices for this animal species. Therefore, water buffalo represents a suitable model to study causative agents of gastroenteritis. In water buffalo, *S*. *enterica* serovar Typhimurium can induce a variety of clinical syndromes with different anatomopathological lesions [1,3]. The severity of the disease can depend on several factors, including host-pathogen interactions, which is highly influenced by the route of infection, the infectious dose, natural or acquired host resistance factors, and the possible presence of other pathogens. Moreover, specific *Salmonella* virulence factors, frequently located on *Salmonella* pathogenicity islands (SPIs), prophage regions or virulence plasmids, play a key role in the pathogenesis of the gastroenteritis [5].

The current study investigated the intestinal contents collected from 248 water buffalo calves affected by gastroenteritis with lethal outcome to: (i) evaluate the prevalence of *Salmonella* spp., and (ii) perform a polyphasic characterization of the collected isolates of *S*. Typhimurium.

## Results and discussion

*Salmonella* spp. were isolated from 25% of the intestinal contents collected from 248 water buffalo calves affected by gastroenteritis with lethal outcome. Positive samples were detected in subjects bred in 37 of 58 farms (inter-herd prevalence, 64%). The *S*. *enterica* serovars most frequently isolated were Typhimurium (n=13), Muenster (n=7) and Give (n=7). Other recovered serovars were: Derby (n=5), 4 Bovismorbificans (n=4), Newport (n=4), monophasic *S*. Typhimurium (B:4,12:i:-; n=3), Blockley (n=2), Meleagridis (n=2), Umbilo (n=2), Altona (n=1), Anatum (n=1), Bredeney (n=1), Enterica (−;i;1,2; n=1), Gaminara (n=1), Haardt (n=1), Hadar (n=1), Infantis (n=1), Isangi (n=1), Kottbus (n=1), London (n=1), Muenchen (n=1), and S.II:41;z;1,5 (n=1). Phage-typing of the *S*. Typhimurium and monophasic Typhimurium strains (Table 1) indicated a variable distribution of phage types among strains with nine different phage types of 13 Typhimurium strains, and three different phage types out of three monophasic Typhimurium strains.

**Table 1:** **Virulotypes and phage types of the*Salmonella*Typhimurium and monophasic*S*. Typhimurium isolates**

**Isolate #**					**Genes**^**a**^							**Genotype #**	**Phage type**
	***gipA***	***gtgB***	***gogB***	***sspH1***	***sodC1***	***gtgE***	***spvC***	***safC***	***csgA***	***pefA***	***agfA***		
*S.* Typhimurium													
16	-	+	+	+	+	+	+	+	+	+	-	1	DT1
92	-	+	+	+	+	+	+	+	+	+	+	2	DT104
112	-	-	-	+	-	-	-	-	+	-	-	3	RDNC
148	+	+	+	+	+	+	-	+	+	-	-	4	DT194
233	-	+	+	-	+	+	+	+	+	+	-	5	DT104
279	-	+	+	-	+	+	+	+	+	+	-	5	U302
107025	-	+	+	-	+	+	+	+	+	-	+	6	RDNC
461	+	+	+	-	+	+	-	+	-	-	-	7	DT208
10606	-	+	+	+	-	-	+	+	+	+	+	10	U302
51789	+	+	+	+	-	+	-	+	+	-	+	8	DT110
55137	+	+	+	+	-	+	-	+	+	-	+	8	DT20
82280	+	+	+	+	-	+	+	+	+	-	+	9	DT110
83528	+	+	+	+	-	+	-	+	+	-	+	8	NT^b^
Freq. (%)	46	92	92	69	54	85	54	92	92	38	54		
monophasic *S.* Typhimurium													
154	-	+	+	+	+	+	-	-	-	-	+	11	DT193
175	-	-	-	+	-	-	-	+	-	-	-	12	U311
188	-	-	-	+	-	-	-	-	+	-	+	13	NT

^a^ The following *loci*: *invA*, *sspH2*, *stfE*, *ipfD*, *bcfC*, *stbD, fimA, avrA, ssaQ, mgtC, siiD, sopB* were present in all the strains; the *sopE* gene was not found in any of these strains.

^b^ NT = not typeable.

This study reports a significant prevalence of *Salmonella* spp. (25%) in diarrheic water buffalo calves, that are more relevant than those reported in previous studies (11 and 0.8%) [3,6]. Moreover, in contrast with bovine species where salmonellosis results primarily associated with serovars Dublin and Typhimurium [5], the extremely variable distribution of the observed serovars confirms the absence of a serovar specifically adapted to water buffalo, as previously suggested [1]. These data provide therefore evidence that *Salmonella*, particularly *S*. Typhimurium, can be potentially considered an important pathogen for this animal species. The definitive phage type 104 (DT104), which has often been associated with multiple-antibiotic-resistant strains with ascertained zoonotic potential and, in many countries, has increased over the past two decades [5], does not seem to be widely spread in water buffalo. Three monophasic *S*. Typhimurium (B:4,12:i:-) isolates were also found that are *S*. Typhimurium lacking phase two flagellar antigens that have a rapid emergence and dissemination in food animals, companion animals, and humans. More significantly, the public health risk posed by these emerging monophasic *S*. Typhimurium strains is considered comparable to that of other epidemic *S*. Typhimurium [7].

**Figure 1:**
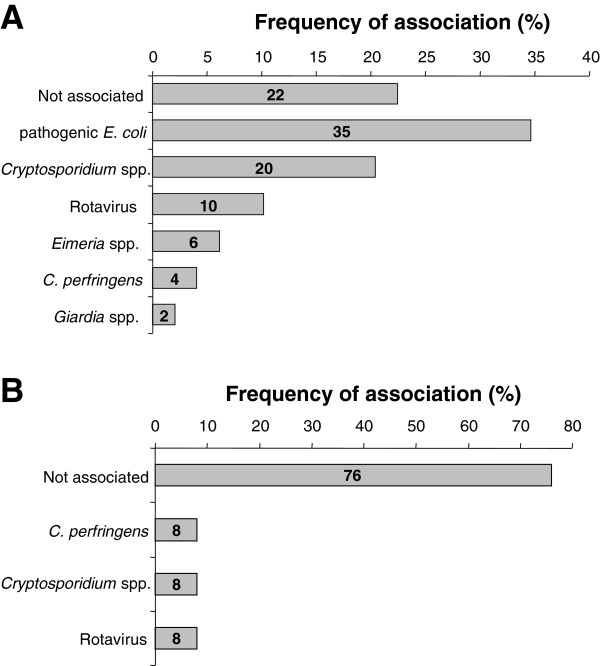
**Frequency of detection of*Salmonella*with other microorganisms.** (**A**) Frequency of association of non-Typhimurium *Salmonella* isolates with microorganisms possibly responsible for gastroenteritis in water buffalo calves. (**B**) Frequency of association of *S*. Typhimurium strains with microorganisms possibly responsible for gastroenteritis in water buffalo calves.

The diagnostic investigation indicated that non-Typhimurium *Salmonella* isolates were detected with at least another potential pathogen in 78% of cases (Figure 1A). In 35% of cases *Salmonella* was linked with pathogenic *Escherichia coli* that were characterized for the presence of virulence factors. Other frequent associations were found with *Cryptosporidium* spp. (20%) and Rotavirus (10%) (Figure 1A). Remarkably, *S*. Typhimurium was never associated with pathogenic *E*. *coli*, while it was isolated sporadically with *Clostridium perfringens* (strain #82280), Rotavirus (strain #107025), and *Cryptosporidium* spp. (strain #112) (Figure 1B). The presence of more pathogens in the same subject might suggest that, as for other animal species [5], diarrhea in water buffalo calves can be characterized by a multifactorial etiology. Data from necroscopic examinations of tissues indicated that the lesions caused by *S*. Typhimurium were characterized by severe damage of the intestine, ranging from congestive to necrotic-ulcerative enterocolitis. In particular, the strains isolated from animals exhibiting the most severe lesions were #16, #92, #233, and #83528. Among these strains, the two DT104 strains were also found, thus supporting the pathogenic role of this phage type. The other *Salmonella* serovars were instead isolated from subjects exhibiting a variety of different lesions, mostly minor lesions confined to the jejunum, and often (78% of cases) associated with other pathogens. Similarly, the monophasic *S*. Typhimurium strains were detected either with Rotavirus (strain #154) or *st*-positive *E*. *coli* (strains #175 and #188). These data confirm the pathogenic potential of the serovar Typhimurium for water buffalo calves. On the other hand, the scarcity of observed lesions and the frequent presence of more than one microorganism in the same subject hamper a clear understanding of the potential pathogenic role of the non-Typhimurium *Salmonella* serovars included in this study.

*S*. Typhimurium and monophasic *S*. Typhimurium strains were further characterized by the molecular detection of 24 genes coding for virulence factors. The genetic characterization (Table 2) included five *loci* (*avrA*, *ssaQ*, *mgtC*, *siiD*, and *sopB*) located on SPI 1–5, respectively [8], eight *loci* (*gipA*, *gtgB*, *sopE*, *sodC1*, *gtgE*, *gogB*, *sspH1*, and *sspH2*) of prophage origin [9-13], the gene *spvC*, located on a virulence plasmid [12], and nine genes (*stfE*, *safC*, *csgA*, *ipfD*, *bcfC*, *stbD*, *pefA*, *fimA*, and *agfA*) coding for bacterial fimbriae, involved in surface adhesion and gut colonization [5]. As a positive control for the PCR assay, amplification of the chromosomal gene *invA* was carried out for each strain. All the *S*. Typhimurium and monophasic Typhimurium isolates displayed the presence of *avrA*, *ssaQ*, *mgtC*, *siiD*, *sopB*, *sspH2*, *stfE*, *ipfD*, *bcfC*, *stbD*, and *fimA* genes, and the absence of the *sopE* gene. Other loci were variably distributed among the strains, with frequency values ranging from 38-92% (Table 1). On the basis of the presence or absence of the 24 loci included in the study, the 13 strains of *S*. Typhimurium were subdivided into 10 different genotypes (Table 1); however, the isolates with identical genotype displayed different phage types suggesting the presence of 13 different strains. Interestingly, the three monophasic *S*. Typhimurium strains exhibited three different genotypes (Table 1).

**Table 2:** ***Salmonella*virulence genes detected by PCR analysis**

**Gene**	**Function**	**Primer sequence (5**^**′**^ – **3**^**′**^**)**	**bp**	**Reference**
*avrA*	Inhibits the proinflammatory, antiapoptotic NF-kappa B pathway	CCTGTATTGTTGAGCGTCTGG	422	[8]
		AGAAGAGCTTCGTTGAATGTCC		
*ssaQ*	Secretion system apparatus protein, component of second T3SS	AATGAGCTGGGTAGGGTGTG	216	This study
		ATGCAACGCTAGCTGATGTG		
*mgtC*	Intramacrophage survival protein	TGACTATCAATGCTCCAGTGAAT	677	[8]
		ATTTACTGGCCGCTATGCTGTTG		
*siiD*	HLYD family secretion protein	GTTCATGGTCAGGGCGTTAT	416	This study
		GCAAGCAATGCGAGTTCTTT		
*sopB*	Translocated effector protein (phosphoinositide phosphatase) via T3SS	TAACGTCAATGGCAAACCAA	334	This study
		CCCTCATAAGCACTGGGAAA		
*gipA*	Peyer’s patch-specific virulence factor	GCAAGCTGTACATGGCAAAG	212	[9]
		GGTATCGGTGACGAACAAAT		
*gogB*	Type III-secreted substrate of the infection process	GCTCATCATGTTACCTCTAT	598	[10]
		AGGTTGGTATTTCCCATGCA		
*sopE*	Translocated T3SS effector protein	CGAGTAAAGACCCCGCATAC	363	[10]
		GAGTCGGCATAGCACACTCA		
*gtgB*	Translocated T3SS effector protein	TGCACGGGGAAAACTACTTC	436	[9]
		TGATGGGCTGAAACATCAAA		
*sspH1*	*Salmonella* secreted protein H1	TGCAGAAAAAGGGGAATACG	246	This study
		GCAGCCTGAAGGTCTGAAAC		
*sspH2*	*Salmonella* secreted protein H2	GCACAACTGGCTGAAGATGA	203	This study
		TTTCCCAGACGGAACATCTC		
*gtgE*	SPI2 type III secreted effector protein	AGGAGGAGTGTAAAGGT	1114	[11]
		GTAGAACTGGTTTATGAC		
*sodC1*	Periplasmmic Cu, Zn-superoxide dismutases	TATTGTCGCTGGTAGCTG	468	[11]
		CAGGTTTATCGGAGTAAT		
*spvC*	Spv region promotes rapid growth and survival within the host	ACTCCTTGCACAACCAAATGCGGA	571	[12]
		TGTCTTCTGCATTTCGCCACCATCA		
*invA*	Enables the bacteria to invade cells	ACAGTGCTCGTTTACGACCTGAAT	244	[12]
		AGACGACTGGTACTGATCGATAAT		
*stfE*	Minor fimbrial subunit of the *Salmonella* Typhi flagella	ATTTGGCAATGTGTTGACGA	185	This study
		TTTGCAGACGGATACCCAAT		
*safC*	Pilin outer membrane usher protein	CTCGCTGTCATTGAACTGGA	158	This study
		CACCGTGTGATGGTGAAGTC		
*csgA*	Major fimbrial subunit of thin curled fimbriae	GGATTCCACGTTGAGCATTT	212	This study
		CGGAGTTTTTAGCGTTCCAC		
*ipfD*	The Ipf fimbrial operon mediates adhesion to Peyer’s patches	TTCCCTCAATACGCAGGAAG	183	This study
		CTCAGGGCTGTGAACTCTCC		
*bcfC*	Bovine colonization factor, fimbrial usher	CAGCTTTTCATGACGCGATA	241	This study
		CAATGTCTCTGGTTGCGAGA		
*stbD*	Stability protein involved in a toxin-antitoxin system and in plasmid stability	GGCTGTAATATTCGCCGGTA	201	This study
		GCACGCCCTATTCCAGTAAA		
*pefA*	Major fimbrial subunit of the plasmid encoded fimbria	ACACGCTGCCAATGAAGTGA	450	[18]
		ACTGCGAAAGATGCCACAGA		
*fimA*	Type 1 major fimbrial unit	CCTTTCTCCATCGTCCTGAA	85	This study
		TGGTGTTATCTGCCTGACCA		
*agfA*	Aggregative fimbria A	GGATTCCACGTTGAGCATTT	312	[18]
		GTTGTTGCCAAAACCAACCT		

The 24 loci-genetic characterization was also extended to the *S*. Muenster and *S*. Give isolates to investigate their pathogenic potential because of their large presence in water buffalo calves. In addition they have already been reported to cause saepticemic salmonellosis in cattle and calves [14,15]. The molecular results (Table 3) indicated that the loci *invA*, *safC*, *bcfC*, *fimA* and *ssaQ* were present in all the strains, the genes *gipA*, *gogB*, *sspH2*, *sodC1*, *gtgE*, *spvC*, *stfE*, *ipfD* and *pefA* were not found in any of these isolates, while the remaining loci were variably distributed, with frequency values ranging from 14-86%. In particular, the prophage genes were scarcely present (2 loci in the Muenster serovar, 1 locus in the Give serovar), the plasmidic *spvC* locus was absent in all the analyzed isolates, while the fimbrial genes and the SPI 1–5 genetic markers were discretely represented (6 loci for the former genes in both serovars, 5 and 4 loci for the latter genes in the serovar Muenster and Give, respectively). Moreover, the molecular profiles allowed to identify 6 different genotypes out of the 7 *S*. Muenster isolates, and 5 different genotypes out of the 7 *S*. Give isolates (Table 3).

**Table 3:** **Virulotypes of the*Salmonella*Muenster and give isolates**

**Isolate #**					**Genes**^**a**^						**Genotype #**
	***gtgB***	***sopE***	***sspH1***	***csgA***	***stbD***	***agfA***	***avrA***	***mgtC***	***siiD***	***sopB***	
*S.* Muenster											
1885	-	+	-	+	+	-	+	+	+	+	1
67	+	+	-	-	-	-	+	-	-	-	2
15228	-	+	-	-	-	-	-	-	-	-	3
66761	-	+	-	-	-	-	-	-	-	-	3
72827	-	+	-	-	-	-	+	-	+	-	4
75822	+	+	-	-	-	-	-	-	-	-	5
66325	-	+	-	+	+	+	+	+	+	+	6
Freq. (%)	29	100	0	29	29	14	57	29	43	29	
*S.* Give											
1139	-	-	-	-	+	-	+	+	+	-	1
364	-	-	+	-	+	-	+	+	+	-	2
18327	-	-	+	-	+	-	+	+	+	-	2
30877	-	-	+	-	+	-	+	-	-	-	3
2670	-	-	+	-	+	-	-	-	-	-	4
100739	-	-	+	+	+	+	+	+	+	-	5
82613	-	-	+	-	+	-	+	+	+	-	2
Freq. (%)	0	0	86	14	100	14	86	71	71	0	

^a^ The following *loci*: *invA*, *safC*, *bcfC*, *fimA* and *ssaQ* were present in all the strains; the genes *gipA*, *gogB*, *sspH2*, *sodC1*, *gtgE*, *spvC*, *stfE*, *ipfD* and *pefA* were not found in any of these strains.

Our data confirm the high variability of the Typhimurium serovar [9,10], mostly related to virulence factors, and highlight the high discriminating potential of the genotyping technique performed. Our data also suggest that monophasic Typhimurium strains are likely to possess a similarly high degree of genetic variability, particularly linked to virulence markers. Moreover, the presence of virulence markers in the isolated strains of monophasic *S*. Typhimurium, *S*. Muenster and *S*. Give could further support their pathogenic potential. The products of the genes included in the virulotyping assay performed here are known to be important during different stages of infection (Table 2). However, the distribution of these factors among the tested strains highlights the complexity and the variety of potential mechanisms used by *Salmonella* to induce disease in the host.

The *avrA*, *ssaQ*, *mgtC*, *siiD*, and *sopB* genes are genetic markers for the presence of the SPI 1–5 in all *S*. Typhimurium strains tested, although their presence does not necessarily implicate the presence of the entire SPI. SPIs are clusters of genes on the chromosome, likely to be horizontally acquired, and variably associated with enhanced invasion and intracellular survival within both phagocytic and non-phagocytic cells. In particular, SPI-5 has been largely associated with the ability to produce enteritis [5]. The *S*. Typhimurium strains included in this study all displayed the presence of the investigated SPI markers. Interestingly, these loci appeared widely distributed also among the serovars Muenster and Give. The *sopE* gene is known to favor the entry of *Salmonella* into host cells and its presence has been correlated with disease in humans [16] and with the epidemic potential of *S*. Typhimurium strains in cattle [17]. This gene was absent in all the *S*. Typhimurium strains included in the present study, while was present in all the *S*. Muenster strains analyzed.

The *pefA* (plasmid encoded fimbria), *agfA* (aggregative fimbria A) and *spvC* (*Salmonella* plasmid of virulence gene C) genes are all located on plasmids [18]. Five *S*. Typhimurium isolates tested in the current study possessed both *pefA* and *spvC*, two isolates were positive for only *spvC*, and three isolates were positive for only *agfA* (Table 1). These results confirm the presence of more than one virulence plasmid among *S*. Typhimurium strains isolated from diarrheic water buffalo calves, and suggest horizontal exchange of virulence factors. However, the loci *pefA* and *spvC* were absent in all the monophasic *S*. Typhimurium, *S*. Muenster and *S*. Give strains tested. Prophage genes are known to account for most of the variability of closely-related *S*. Typhimurium strains. Moreover, lysogenic bacteriophages promote changes in the composition of genomic DNA often altering the phenotype of the host [9,10]. The prophage virulence genes included in this study exhibited a variable distribution among the isolates tested, thus suggesting synergistic and/or redundant effects of these loci on the pathogenicity of *Salmonella*, likely contributing to the phenotypic variability of this pathogen. These loci were mostly present in *S*. Typhimurium and monophasic *S*. Typhimurium rather than in *S*. Muenster and *S*. Give isolates. Fimbrial genes appeared widely distributed among all the serovars tested, particularly in *S*. Typhimurium strains, with frequency values ≥92%, except for the plasmid-borne *pefA* and *agfA* genes (with frequency values of 38% and 54%, respectively). These data are consistent with the essential functions of adhesion factors for the attachment and internalization processes that occur during pathogenesis.

To better characterize *in vivo* virulence, three strains representative of all *S*. Typhimurium isolates were chosen to perform mixed infections in mice. Animal experiments included the two strains exhibiting the highest and the lowest number of virulence factors (strains #92 and #112, respectively), and strain #16, carrying the same virulotype as strain #92, but that does not harbor the *agfA* locus (Table 1). In the competition assay, strain #92 outcompeted both strains #112 and #16 (CI 0.004; *P*<0.001, and CI 0.031; *P*<0.001, respectively). These results were confirmed in a gastrointestinal mouse model of infection, which better resembles the clinical form of salmonellosis in livestock. Using oral inoculation, in the competition assay, again strain #92 outcompeted both strains #112 and #16 (CI 0.009; *P*<0.001, and CI 0.186; *P*<0.01, respectively). Our data indicate that among those strains included in the experiment, strain #92 was the most virulent in mice. These competition assays in mice suggest a key role of the *agfA* gene coding for a thin aggregative fimbria involved in the colonization of host intestinal epithelial cells by attachment to glycoprotein or glycolipid receptors on epithelial cell surfaces. Indeed, the strain which was more virulent in *in vivo* experiments was characterized by a high number of virulence factors and by the presence of the *agfA* locus. Moreover, it was isolated from one of the subjects with necrotic-ulcerative enterocolitis.

The presence of this type of fimbria has been reported in clinical human and animal isolates of *Salmonella*[19,20]. The data presented here suggest that *agfA* might increase bacterial pathogenicity. Nevertheless, we cannot reject the hypothesis that the mouse model chosen for *in vivo* experiments could have influenced the virulence phenotype of the tested strains originally isolated from water buffalo calves. Therefore, future studies will be necessary to exclude the possibility that the phenotypic differences observed among the tested Salmonellae are dependent on the animal model or on other virulence factors not included in this study. However, *in vivo* experiments carried out in mouse models represent a good preliminary source of information on the expression of traits associated with pathogenicity of *Salmonella* in mammalian species.

## Conclusions

This study showed a significant (25%) prevalence of *Salmonella* spp. in water buffalo calves affected by gastroenteritis with lethal outcome. However, our results did not indicate the existence of a *Salmonella* serovar specifically adapted to water buffalo and highlighted that *S*. Typhimurium is the most frequently found serovar. The molecular and phenotypic characterization of the *S*. Typhimurium isolates provided evidence that within this serovar there are different pathotypes potentially responsible for different clinical syndromes, therefore requiring prophylaxis protocols including the use of specific vaccines for the effective control of salmonellosis in water buffalo calves and possible contamination of the food chain.

## Methods

### Bacterial strains and diagnostic methods

This study was carried out in the Campania region, Southern Italy, during 2008–2009, using samples taken from 248 water buffalo calves bred in 58 different farms. The animals were aged between 1–12 weeks old and were all affected by gastroenteritis with lethal outcome. During necropsy, the intestinal lesions were evaluated and the intestinal content of the involved sections was collected and tested for the presence of *Salmonella* spp. In addition, the presence of *E*. *coli*, *Eimeria* spp., *Cryptosporidium* spp., *Giardia* spp., Coronavirus, Rotavirus, and *C*. *perfringens* were also determined to investigate their association with *Salmonella* spp.

The isolation of *Salmonella* spp. was performed according to ISO 6579:2002 [21]. The isolated *Salmonella* spp. were serotyped according to the Kaufmann-White scheme [22]. Phage-typing of the isolated *S*. Typhimurium strains was performed by the Italian National Reference Centre for Salmonellosis (Istituto Zooprofilattico Sperimentale delle Venezie).

The presence of Rotavirus and Coronavirus was detected by polymerase chain reaction (PCR) amplification [23,24]. *Cryptosporidium* spp. and *Giardia* spp. antigens were detected by chromatographic immunoassay (Oxoid, Basingstoke, UK). The presence of *Eimeria* spp. was examined by flotation technique using saturated saline [25]. *E*. *coli* and *C*. *perfringens* were isolated according to the protocol reported by Quinn *et al*. [2]. *E*. *coli* hemolytic activity was evaluated by growing colonies on blood agar base, while virulence factors (*lt*-heat-labile toxin, *st*-heat-stable toxin, *stx1*-Shiga toxin 1, *stx2*-Shiga-toxin 2, *eae*-intimin, *cnf*-cytotoxic necrotizing factor, and *cdt*-cytolethal distending toxin) were detected by molecular assays, as previously reported [26-28].

### DNA extraction and molecular assays

Bacterial DNA was extracted from 1 mL of overnight cultures using Chelex 100 Resin (BioRad, Hercules, CA) and used as the template for the PCR detection of genes listed in Table 2, as described previously [8-13,18]. The primers used to amplify the genes *sspH1*, *sspH2*, *ssaQ*, *sopB*, *siiD*, *stfE*, *safC*, *csgA*, *ipfD*, *bcfC*, *stbD*, and *fimA* were designed using the Primer3 software (version 0.4.0; http://frodo.wi.mit.edu/), and PCR was performed in a final volume of 25 μL containing HotStar Taq Master Mix (Qiagen, Valencia, CA) 1×, 0.4 μM each primer and 1 μL of extracted DNA. The thermal profile included an initial denaturation step at 95°C for 15 min, followed by 35 cycles at 95°C for 30 s, 58°C for 30 s, and 72°C for 1 min, and a final extension step at 72°C for 5 min. Amplification products were visualized under ultraviolet (UV) light after electrophoresis on 3% agarose gels and staining with SYBRsafe (Invitrogen, Carlsbad, CA).

### Competition assays in mice

Groups of five age matched (8–10 weeks old) female BALB/c mice used in this study were purchased from Charles River (Calco, Italy). Three strains (*S*. Typhimurium #16, *S*. Typhimurium #92, *S*. Typhimurium #12), representative of the 13 genotypically characterized *S*. Typhimurium isolates, were selected for an *in vivo* analysis of virulence by using the Competitive Index (CI) resulting from mixed infections [29]. In particular, two strains were selected that exhibited the highest and lowest number of virulence factors (strains #92 and #112, respectively), and strain #16, carrying the same virulotype as strain #92, but without the locus *agfA* (Table 1).

Bacteria were grown overnight at 37°C in Brain Heart Infusion medium (Oxoid, Basingstoke, UK), washed, and diluted in sterile saline. Cultures were alternatively combined in a mixture of equivalent numbers (1:1 ratio) of two of the three selected strains (input). Mice were inoculated intraperitoneally (IP) with a dose of 2×10^4^ bacteria or received 20 mg of streptomycin orally (200 μL of sterile solution or sterile saline) 24 h prior of being intragastrically administered with 2×10^7^ bacteria. The number of colony-forming units (CFU) contained in the inocula were confirmed by plating serial dilutions and counting colony growth. At 4 (IP) or 7 (os) days after infection, mice were sacrificed, spleens were aseptically removed, and bacteria were counted by plating serial dilutions (output). The ratio of two strains in the input and in the output was evaluated by picking and transferring 200 colonies on selective plates. Antibiotics used were streptomycin and sulfonamide, for which strain 92 and strains 16 or 112 were naturally resistant. The CI was calculated using the formula: CI = output (strain A/strain B)/inoculum (strain A/strain B). Statistical differences between outputs and inputs were determined by Student’s *t* test. All animal handling and sampling procedures were performed under the conditions of the local ethics committee meeting the requirements of Italian legislation.

## Competing interests

The authors declare that they have no competing interests.

## Authors’ contributions

GB carried out the molecular genetic studies and drafted the manuscript. MGL contributed to the molecular analysis and the isolation and phenotypic characterization of the strains. MP designed and interpreted the results of the *in vivo* assays. MRC carried out the isolation and phenotypic characterization of the strains. CG participated in the design of the *in vivo* assays and performed the statistical analysis. SA and AB carried out the *in vivo* assays and participated in the phenotypic characterization of the strains. DE contributed to the design of the molecular assays, the interpretation of the genotyping results and critical preparation of part of the manuscript. PP participated in the conception, design, and coordination of the study. GG conceived the study, and participated in its design and coordination, and helped to draft the manuscript. All authors read and approved the final manuscript.
